# Association between HIV replication and serum leptin levels: an observational study of a cohort of HIV-1-infected South African women

**DOI:** 10.1186/1758-2652-13-33

**Published:** 2010-09-07

**Authors:** Livio Azzoni, Nigel J Crowther, Cynthia Firnhaber, Andrea S Foulkes, Xiangfan Yin, Deborah Glencross, Robert Gross, Mitch D Kaplan, Emmanouil Papasavvas, Doreen Schulze, Wendy Stevens, Tessa van der Merwe, Rita Waisberg, Ian Sanne, Luis J Montaner

**Affiliations:** 1 The Wistar Institute, Philadelphia, PA, USA; 2 Department of Chemical Pathology, National Health Laboratory Service and University of the Witwatersrand, Johannesburg, South Africa; 3 Clinical HIV Research Unit, University of the Witwatersrand, Johannesburg, South Africa; 4 University of Massachusetts, Amherst, USA; 5 Department of Hematology and Molecular Medicine, National Health Laboratory Service and University of the Witwatersrand, Johannesburg, South Africa; 6 Division of Infectious Diseases/Department of Medicine and Center for Clinical Epidemiology and Biostatistics, University of Pennsylvania School of Medicine, Philadelphia, PA, USA; 7 Rosebank Hospital, Johannesburg, South Africa; 8 University of Pretoria and Network Healthcare Holdings, Pretoria, South Africa

## Abstract

**Background:**

Advanced HIV infection can result in lipoatrophy and wasting, even in the absence of ongoing opportunistic infections, suggesting that HIV may directly affect adipose tissue amount and distribution.

**Methods:**

We assessed the relationship of fat (measured using anthropometry, DEXA, MRI scans) or markers related to glucose and lipid metabolism with viral load in a cross-sectional sample of 83 antiretroviral-naïve HIV-1-infected South African women. A multivariable linear model was fitted to log_10_VL to assess the combined effect of these variables.

**Results:**

In addition to higher T cell activation, women with viral load greater than the population median had lower waist circumference, body mass index and subcutaneous abdominal fat, as well as lower serum leptin. We demonstrate that leptin serum levels are inversely associated with viral replication, independent of the amount of adipose tissue. This association is maintained after adjusting for multiple variables associated with disease progression (i.e., cellular activation and innate immunity effector levels).

**Conclusions:**

Our results demonstrate that serum leptin levels are inversely associated with viral replication, independent of disease progression: we postulate that leptin may affect viral replication.

## Background

Progressive HIV infection [1,2] leads to loss of body fat, altering subjects' appearance and resulting in social stigma and negative body image, particularly in women [3]. Caloric intake and adiposity are regulated by adipokines, neurotransmitters, hormones and their receptors that modulate appetite, fat metabolism and energy homeostasis [4]. Leptin is produced by adipocytes, and contributes to appetite reduction via a negative hypothalamic feedback [5], as well as exerting immunomodulatory activity (e.g., inhibition of cell proliferation and promotion of type 1 adaptive responses [6-8]).

A correlation between adipose tissue and leptin is maintained in antiretroviral-treated HIV-infected individuals, independent of viremic status or disease stage [9-12], even though leptin receptor expression and phosphorylation are increased in peripheral blood mononuclear cells [13]. A number of factors (chronic fever, increased TNF-α and IL-6 levels, opportunistic infections and other secondary causes [1,14,15]) have been considered to contribute to fat loss in advanced disease; however, a direct link to viral replication has not been established.

Women account for more than 50% of HIV-1 prevalence worldwide [16]. Here, we explore the relationship between viral load, cellular activation, innate immunity effector levels and adipose tissue-related measures in a cohort of antiretroviral therapy (ART)-naïve South African women.

## Methods

### Study subjects

A cross-sectional convenience sample of 83 ART-naïve, HIV-1 infected women without evidence of prior or ongoing opportunistic infection was enrolled at the Clinical Research HIV-1 Unit, Themba Lethu Clinic (University of the Witwatersrand, Johannesburg, South Africa). Screening CD4+ T cell count was 200-350 cells/mm^3^. Medical history was obtained from the clinic record and by interview. Informed consent was obtained from all participants as per University of the Witwatersrand Ethics Committee and Wistar Institute IRB-approved study protocol.

### Flow cytometry

CD4+ T cell counts were assessed using the single platform method described by Scott and Glencross [17]. Expression of lineage, differentiation and activation markers on CD3+ T cells (CD4, CD8, CD38, CD95, HLA-DR, CD28), CD3- NK cells (CD16, CD56, HLA-DR) and Lin-1^- ^Dendritic cells (Lin-1, HLA-DR, CD123, CD11c) was assessed on whole peripheral blood using lyophilized mAb panels (BD Biosciences, Palo Alto, CA). Samples were tested using a Faxcalibur Analyzer, followed by analysis using CellQuest software (BD Biosciences).

### Adipose tissue measurements

Body mass index (BMI) was calculated as weight (kg)/height (m)^2^. Dual Energy X-ray Absorptiometry (DEXA) scans were performed using a Hologic QDR-2000 scanner, assessing limb and trunk fat and lean mass. Bone mineral density (g/cm^2^) was also measured.

Magnetic resonance imaging (MRI) scans were performed using a Toshiba Flexart 0.5 T; a single L4-L5 axial section was analyzed. Variables collected were sagittal diameter, visceral, subcutaneous abdominal and perirenal fat. The analysis was conducted using V3.51*R553 software.

### Clinical laboratory testing

Serum from fasting blood draws was tested for:

• *Leptin*: ELISA, BioVendor Laboratory Medicine, Inc, Czech Republic

• *High density lipoprotein (HDL)- and low density lipoprotein (LDL)-associated cholesterol, triglycerides, glucose*: Roche Integra analyzer 400, Roche Diagnostics, Mannheim, Germany

• *Insulin*: Immulite 1000 analyzer Diagnostics Corp, Los Angeles. CA

• *Proinsulin*: ELISA, Dako-Cytomation Ltd (UK)

• *Free fatty acids (FFA)*: half-micro test, Roche Diagnostics, Mannheim, Germany

• *HIV-1 infection (confirmation)*: rapid antibody testing and/or Ultra-sensitive (US) PCR, (Roche COBAS Ampliprep/COBAS Amplicor v1.5 methods)

• *Homeostatic Model Assessment for insulin resistance (HOMA2-IR) *was determined using the HOMA2 calculator, v. 2.2, from the Diabetes Trials Unit, University of Oxford, GB http://www.dtu.ox.ac.uk/homa.

### Statistical analysis

Study subjects were divided into two groups: Group 1 (low viral load, LVL): 41 subjects, viral load (VL) < sample median; and Group 2 (high viral load, HVL): 42 subjects, VL ≥ sample median. Differences in means between groups for each of the variables listed in Table 1 were tested using two-sample t-tests. Corresponding p-values and q-values based on the positive false discovery rate (FDR) are reported; q-values of less than 0.2 were considered meaningful. Pairwise correlations were tested by determining the Pearson correlation coefficient (r).

**Table 1:** Expression of metabolic, anthropometric and immunologic markers in high and low viral load subjects

		All subjects (n = 83)			VL ≥ median (n = 42)			VL < median (n = 41)				
			
Variables	Units	N	Mean	SD	N	Mean	SD	N	Mean	SD	unadjusted p *	FDR q**
Age	years	83	34.5	8.2	42	34.7	8.4	41	34.3	8.2	0.85	0.86
log_10_VL	c/ml	83	4.5	0.8	42	5.2	0.3	41	3.9	0.6	n/a	n/a
Immunology markers:												
CD4+ T cell count	cells/mm^3^	83	260.8	53.9	42	249.0	50.7	41	272.9	54.9	0.04	0.14
CD8+ T cell count	cells/mm^3^	59	814.0	484.0	29	874.6	560.8	30	755.4	397.1	0.35	0.51
% CD38^+ ^CD4+ T cells	% of CD4	60	75.5	11.3	30	79.0	9.0	30	72.0	12.3	0.02	0.1
% CD38+ CD8+ T cells	% of CD8	60	87.0	11.2	30	91.1	7.8	30	82.8	12.6	0.0034	0.1
% HLA-DR+ CD4^+ ^T cells	% of CD4	60	26.25	9.71	30	29.5	10.04	30	23.0	8.3	0.0090	0.1
% HLA-DR+ CD8^+ ^T cells	% of CD8	60	56.4	14.3	30	59.6	14.6	30	53.2	13.4	0.08	0.1
% CD95+ CD4+ T cells	% of CD4	60	78.5	10.9	30	80.6	10.9	30	76.4	10.6	0.13	0.28
% CD95+ CD8+ T cells	% of CD8	60	60.6	7.2	30	60.4	6.1	30	60.8	8.3	0.84	0.86
% CD28+ CD4^+^+T cells	% of CD4	60	80.7	11.1	30	80.1	10.7	30	81.2	11.7	0.70	0.82
% CD28+ CD8+ T cells	% of CD8	60	30.8	9.3	30	30.3	9.7	30	31.3	9.1	0.68	0.82
Total NK cells	cells/mm^3^	59	12.9	10.2	29	14.4	12.9	30	11.5	6.6	0.28	0.46
% CD56+/CD16+ cells	% of NK	60	54.9	20.1	30	50.4	18.8	30	59.4	20.6	0.08	0.1
% CD56+/CD16^- ^NK cells	% of NK	60	22.1	16.2	30	23.4	17.6	30	20.9	14.9	0.55	0.74
% HLA-DR^+ ^total NK cells	% of NK	60	19.9	16.8	30	22.2	17.9	30	17.6	15.6	0.29	0.46
% HLA-DR+ CD56+/CD16+ NK cells	% of NK	60	23.0	20.8	30	26.2	22.7	30	19.7	18.6	0.23	0.4
Plasmacytoid dendritic cells	cells/mm^3^	58	0.2	0.1	28	0.1	0.1	30	0.2	0.1	0.18	0.37
Myeloid dendritic cells	cells/mm^3^	59	0.5	0.8	29	0.5	0.6	30	0.5	1.0	0.86	0.86
Adipose tissue-associated variables:												
Body mass index (BMI)	kg/m^2^	82	27.4	7.1	41	25.6	6.4	41	29.2	7.4	0.02	0.1
Waist circumference	cm	83	83.9	14.9	42	80.2	13.4	41	87.6	15.5	0.02	0.1
Subcutaneous abdominal fat (MRI)	cm^2^	81	338.8	195.9	42	294.2	185.9	39	386.9	197.3	0.03	0.12
Visceral abdominal fat (MRI)	cm^2^	81	51.5	30.4	42	47.3	28.8	39	56.1	31.9	0.20	0.37
Perirenal hilar fat (MRI)	cm^2^	81	5.7	3.1	42	5.5	3.4	39	6.0	2.8	0.40	0.56
Total fat mass (DEXA)	kg	80	26.2	12.2	41	24.1	11.9	39	28.5	12.2	0.11	0.28
Total lean mass (DEXA)	kg	80	40.9	5.9	41	39.8	54.4	39	42.1	6.1	0.09	0.26
Leg fat mass (DEXA)	kg	80	11.6	5.6	41	10.8	4.5	39	12.5	6.5	0.20	0.37
Trunk fat mass (DEXA)	kg	80	10.4	6.1	41	9.4	6	39	11.5	6	0.03	0.12
Weight	kg	83	70.2	17.9	42	67.2	17.1	41	73.2	18.3	0.12	0.28
Reported weight loss	% true	83	38	n/a	42	40	n/a	41	36.7	n/a	0.86***	0.86
Leptin	ng/ml	66	28.3	17.6	33	23.4	17.7	33	33.3	16.3	0.02	0.1
Triglycerides	mmol/l	77	0.9	0.4	38	0.9	0.3	39	0.9	0.4	0.57	0.74
LDL-associated cholesterol	mmol/l	77	2.3	0.8	38	2.2	0.6	39	2.3	0.9	0.62	0.78
Free fatty acids	mmol/l	66	0.5	0.2	33	0.5	0.2	33	0.4	0.3	0.31	0.47
Glucose	mmol/l	74	4.3	0.4	37	4.3	0.4	37	4.3	0.4	0.75	0.85
HOMA2-IR	n/a	67	0.9	0.5	32	0.8	0.4	35	1.0	0.6	0.10	0.27

* based on two-sample t-test

** false discovery rate. q values < 0.2 were considered significant

*** Chi square test

The effect of selected variables on log_10_VL (univariate analysis) was assessed by fitting a linear model using the least square method. Additionally, a multivariable linear model was fitted to log_10_VL using a step-wise approach based on likelihood ratio tests [18]. For the final model, Wald test statistics with p-values < 0.1 were considered relevant. Goodness-of-fit was evaluated based on the adjusted coefficient of determination (R^2^): briefly, variables were added individually to the model, and those variables that did not lead to an increase of R^2 ^were rejected. All statistical tests were performed using r vers. 2.10.0 ("the r Foundation for Statistical Computing", http://www.r-project.org/).

## Results

### Cohort clinical and immunological characterization

Eighty-three HIV-1-infected, ART-naïve women, aged 19 to 55 (mean age 34.5 years, see Table 1), and of black (n = 81) or mixed race (n = 2) were studied. The cohort median serum HIV RNA (VL) was 60,400 copies of HIV RNA/ml (log_10_VL = 4.78; IQR = 1.21). Based on BMI, our cohort was composed of three underweight (BMI < 18.5 kg/m^2^), 33 normal weight (BMI 18.5-24.9 kg/m^2^), 26 overweight subjects (BMI 25-29.9 kg/m^2^) and 20 obese subjects (BMI > 30 kg/m^2^), in keeping with prior reports on similar South African cohorts [19]. The cohort mean CD4+ T cell count was 260 cells/mm^3^, indicative of moderately advanced disease. Due to enrolment restrictions of 200-350 CD4+ T cell count/mm^3^, entry CD4+ T cell counts were only slightly lower in HVL (Table 1) than LVL subjects. However, confirming previous reports [20,21], cellular activation was higher in HVL subjects, as assessed by the expression of CD38 on CD4+ and CD8+ T cells, and of HLA-DR on CD4+ T cells (Table 1). Likewise, we did not detect significant differences in the levels of mature or activated NK cells, or of plasmacytoid (PDC) or myeloid (MDC) dendritic cells.

### Association of viral load with adipose tissue

HVL women had lower BMI and waist circumference than LVL women (Table 1). Direct MRI assessments of abdominal fat content confirmed that HVL subjects had considerably less subcutaneous abdominal fat than LVL women, but similar amounts of visceral and perirenal fat (Table 1). HVL women also had slightly lower DEXA scan-based fat and lean mass measurements than LVL women, but only trunk fat mass reached the level of significance, confirming that the difference in adipose tissue between HVL and LVL women is due to differential representation of central fat. Bone density was similar in the two groups (not shown).

We tested a number of markers associated with fat and glucose regulation (Table 1); of all the indicators assessed, only leptin levels were significantly lower in HVL subjects.

**Figure 1:**
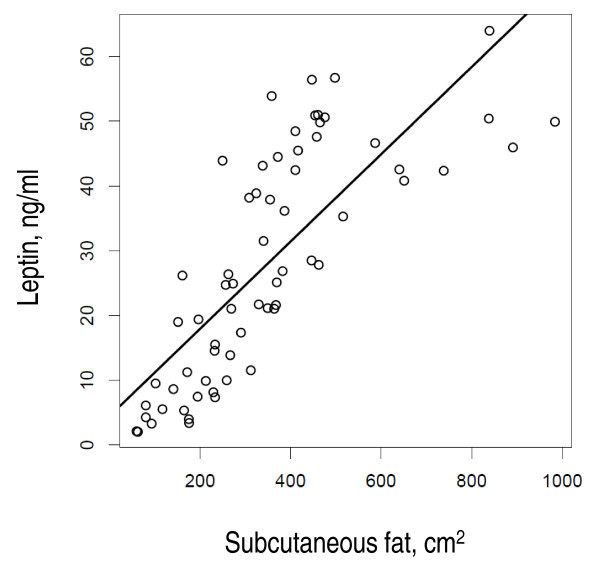
**Association of serum leptin levels with subcutaneous fat**. Linear regression modelling of the association between observed serum leptin levels and MRI-assessed subcutaneous fat area. Circles represent individual observations. P < 0.0001; adjusted R^2 ^= 0.5983.

As expected, leptin levels were directly correlated with BMI (r = 0.6991; p < 0.0001), MRI-measured subcutaneous (Figure 1) or visceral abdominal fat (r = 0.7755 and 0.5417, respectively, both p < 0.0001) or DEXA-based total fat mass, (r = 0.7637; p < 0.0001)].

Unlike direct measures of adiposity, serum lipids and glucose were similar in HVL and LVL women.

Visceral fat was positively associated with levels of insulin (r = 0.3861, p = 0.0010), C-peptide (r = 0.5331, p < 0.0001) and HOMA2-IR (r = 0.2774, p = 0.0241), but not proinsulin/insulin ratio; similar results were obtained for subcutaneous abdominal fat. Reported recent weight loss rates and free fatty acids (FFA) levels were similar in both groups (Table 1), and FFA showed no association with the amount of adipose tissue (r = 0.1084, p = 0.3868).

### Modelling of leptin levels as a predictor of viral load, independent of fat accumulation

**Figure 2:**
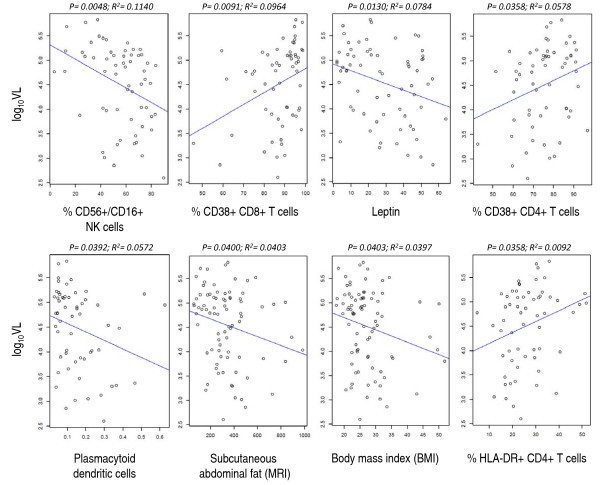
**Association of metabolic and immunologic parameters with HIV viral load**. Linear regression modelling of the association between log_10 _serum viral load and significantly associated variables; individual p values and adjusted R^2 ^for the associations are indicated. Circles represent individual observations.

To determine the relationship between metabolic parameters and viral replication, we first assessed the relationship of all individual variables (see Table 1) with log_10 _VL by fitting a linear model using all available data points (i.e., no censoring of subjects missing individual variable measurements). As illustrated in Table 2 and Figure 2, of the 35 variables tested, only eight had a significant (p < 0.05) effect on log_10_VL based on univariate analysis. As expected, the direction of this effect was positive for parameters associated with activation (CD4+ T cells expressing CD38 or HLA-DR, CD8+ T cells expressing CD38), indicating that subjects with higher viral load also have higher cellular activation. Conversely, a negative effect was observed for PDC counts, mature NK cell frequency, as well as BMI, subcutaneous abdominal fat and leptin (Figure 2), supporting the observations of differences between HVL and LVL women, as we have described. Importantly, the negative association between leptin serum levels and log_10_VL was maintained (n = 65; effect estimate = -0.0186653; p = 0.0289), even after adjusting for subcutaneous abdominal fat area in multivariate analysis, supporting a direct association between leptin levels and viral replication, independent of the amount of adipose tissue.

**Table 2:** Variable association with log_10_VL, univariate analysis*

Predictor	n	Effect estimate	SE	Pr(>|t|)	Adjusted R^2^
% CD56+/CD16+ NK cells	60	-0.005	0.005	0.0048	0.1140
% HLA-DR+ CD4+ T cells	60	0.020	0.009	0.0358	0.0092
% CD38+ CD4+ T cells	60	0.020	0.009	0.0358	0.0578
% CD38+ CD8+ T cells	60	0.025	0.009	0.0091	0.0964
Plasmacytoid dendritic cells	58	-1.718	0.814	0.0392	0.0572
Leptin	66	-0.003	0.005	0.0130	0.0784
Subcutaneous abdominal fat (MRI)	81	-0.001	0.0004	0.0400	0.0403
Body mass index (BMI)	82	-0.025	0.012	0.0403	0.0397

* linear model fitting, least squares method

The eight variables with univariate association to log_10_VL were further tested in a subset of subjects with complete datasets (no missing variable measurements; n = 45). Based on their ability to improve the model predictivity (as assessed by testing the model likelihood ratio), five significant variables were selected to build the additive model: the estimate terms for the model and corresponding test statistics are provided in Table 3. Leptin levels, mature NK frequency and PDC count have a negative effect on log_10_VL that were significant at the 10% level, whereas the positive effect of the expression of CD38 on CD8+ T and HLA-DR on CD4+ T cells was not significant. Taken together, our analysis indicates that leptin levels, together with mature NK and PDC frequency, remain negatively associated with log_10_VL after adjustment for multiple metabolic and activation parameters, suggesting an independent association.

**Table 3:** Effect* of selected variables on viral load: multivariate analysis

Predictor	Effect estimate	SE	T value	Pr (>|t|)
Intercept	3.896921	0.839040	4.644	<0.0001
% CD56+/CD16+ NK cells	-0.009494	0.005328	-1.782	0.0826
Leptin	-0.011514	0.005597	-2.057	0.0464
Plasmacytoid dendritic cells	-2.801874	1.147009	-2.443	0.0192
% HLA-DR+ CD4+ T cells	0.017391	0.010856	1.602	0.1172
% CD38+ CD8+ T cells	0.016283	0.009788	1.664	0.1042

Residual standard error: 0.6274 on 39 degrees of freedom

Multiple R-squared: 0.4301, Adjusted R-squared: 0.357

F-statistic: 5.877 on 5 and 39 DF, p-value: 0.0003816

* The final model is an additive model with main effects for each of the terms

## Discussion

We show for the first time that leptin levels are associated with viral load after adjusting for fat measurements. Body fat changes in HIV-infected individuals have been the subject of a number of studies, many of which have focused on ART-associated lipodistrophy [22-26]. Yet to our knowledge, no study has directly sought to determine the relationship between fat, leptin and HIV replication. Importantly, since opportunistic infections (OIs), in association with lower CD4+ T cell count, might also contribute to low adiposity in chronic HIV infection (via LPS-induced TNF, limited food intake, malabsorption, etc.), we chose to study OI-free, ART-naïve women within a narrow CD4+ T cell count range (200-350 CD4+ T cell count/mm^3 ^at screening); however, a contribution of prior or subclinical OIs and other potential confounding factors cannot be categorically excluded.

The narrowness of the cohort's CD4+ T cell count range (which limits the confounding effects of this variable) might explain the observed lack of a significant effect of CD4+ T cell count levels on VL, which has been consistently reported in larger, unrestricted cohorts [27]. However, as expected, in our cohort, viral load was positively associated with immune activation (CD38 and HLA-DR expression) and negatively associated with the frequency of innate immunity effectors (PDC and mature NK cells) in peripheral blood, confirming prior observations [28-32].

HVL women showed lower BMI, waist circumference and subcutaneous abdominal fat than LVL women, with a significant negative association between VL and several measures of central fat. Leptin levels observed in LVL women were similar to those observed in a cohort of 50 healthy women with similar age, ethnicity and provenance (median BMI 27 kg/m^2^, IQR 10.85; median serum leptin 36.15 pg/ml, IQR = 38.95, N Crowther, unpublished results).

As predicted by the fact that leptin is mainly produced by subcutaneous fat adipocytes [33], this adipokine was also lower in HVL women, and its levels are inversely correlated to viral replication. Interestingly, however, HOMA2-IR, another marker usually highly correlated with adiposity, presented no association with VL in our cohort, and was only weakly associated with leptin levels (p = 0.0751). Taken together, these results indicate that: (a) adipose tissue is associated with both leptin levels and insulin resistance; and (b) only leptin levels are inversely associated with viral replication, suggesting the hypothesis that the inverse relationship between viral replication and leptin levels may be independent of the amount of adipose tissue. Formal testing of this hypothesis in a multivariate model demonstrated that the effect of leptin is in fact independent of direct measures of fat (e.g., subcutaneous fat area).

With the exception of leptin, serum molecules associated with adipose tissue and obesity (e.g., total or LDL cholesterol, triglycerides, glucose, insulin) did not independently correlate with VL in our cohort, in contrast with prior reports that VL could predict BMI in HIV-infected women [2], and correlate negatively with LDL and HDL cholesterol and positively with triglycerides, but not with insulin or glucose levels [34,35]. Indeed, we confirmed the selection of leptin over any other adipose tissue measure that appeared to be individually correlated with log_10_VL by introducing BMI or subcutaneous abdominal fat in a multivariate model with leptin: neither carried a significant independent association (p = 0.3667 and 0.884, respectively), and both actually resulted in making our model less accurate by reducing the adjusted R^2 ^(0.063 and 0.05, as compared to 0.066 for leptin alone).

Therefore, among measures and correlates of adipose tissue, leptin remained the variable best associated with viral replication, suggesting the possibility that leptin may play a role in the observed significant association between fat and viral replication. Interestingly, prior studies (FRAM cohort [36]) testing whether viral replication was associated with serum adipokines did not evidence a significant association between adiponectin and leptin levels and HIV viral load: the fact that our cohort is composed only of ART-naïve women with a narrow CD4 range, and possibly the high levels of adiposity in our cohort, might contribute to this discordance.

Based on leptin's known immunomodulatory activity [6-8,13,37,38], it is interesting to speculate that in HVL women, lower leptin levels may contribute to chronic immune activation, in keeping with the observed increased expression of CD38 and HLA-DR in T cell subsets. Obesity is associated with chronic low-level inflammation and high levels of TNF-α [39], which is produced by subcutaneous adipocytes [40]. This condition would be expected to promote viral replication since TNF-α promotes HIV replication via NFκB activation [41].

The negative association between leptin and viral replication that we report here suggests that leptin, which is produced by adipocytes in response to exposure to TNF-α[42], may be part of a negative regulatory feedback that attenuates the pro-inflammatory and pro-replicative effects of TNF-α. Interestingly, this effect is likely lost in chronic inflammatory conditions where a negative association between TNF-α and leptin production has been observed [43,44]. Conversely, increased body fat may attenuate viral load via the effects of other mediators (e.g., MIP-1α) known to suppress HIV infection [45]). Another potential mechanism could be that high viral replication and cellular activation may result in chronic inflammation, affecting adipocytes and causing lipoatrophy and lower leptin levels.

The interpretation of these results should be qualified in light of the cross-sectional design of the study, which does not allow the interpretation of cause-effect relationships in the variables studied. Further longitudinal studies focusing on these factors will be required to determine whether fat changes directly contribute to alterations in viral replication via adipocyte-mediated leptin secretion.

## Conclusions

Our data provide the first demonstration of a relationship between VL and leptin in African women and suggest that lower leptin levels associated with the loss of adipose tissue may contribute to disease progression.

## Abbreviations

ART: antiretroviral therapy; BMI: body mass index; DEXA: dual energy X-ray absorptiometry; FDR: false discovery rate; FFA: free fatty acids; HDL: high density lipoprotein; HOMA2-IR: homeostatic assessment for insulin resistance; HVL: high viral load; LDL: low density lipoprotein; LVL: low viral load; MDC: myeloid dendritic cells; MRI: magnetic resonance imaging; OI: opportunistic infection; PDC: plasmacytoid dendritic cells; VL: viral load;

## Competing interests

The authors declare that they have no competing interests.

## Authors' contributions

LA was responsible for study design, data management, critical analysis, manuscript and illustration preparation. NJC undertook leptin and lipid assessment, critical analysis, data discussion and manuscript preparation. CF undertook clinical coordination, patient interaction, data discussion and manuscript preparation. ASF undertook statistical analysis, data discussion and manuscript preparation. XY undertook statistical analysis, data discussion and manuscript revision. DG undertook flow cytometry analysis, CD4+ T cell count assessment, data discussion and manuscript preparation. RG undertook statistical and epidemiology analysis, data discussion and manuscript preparation. MDK undertook DEXA scan and MRI performance, DEXA data analysis, data discussion and manuscript preparation. EP undertook data discussion and manuscript revision. DS undertook database management, data discussion and manuscript revision. WS undertook clinical laboratory test performance, data discussion and manuscript preparation. TvdM undertook MRI data analysis, data discussion and manuscript revision. RW undertook leptin and lipid assessment, critical analysis, data discussion and manuscript revision. IS undertook clinical site supervision, clinical coordination, patient interaction, data discussion and manuscript preparation. LJM undertook study design, immunology laboratory supervision, critical analysis and manuscript preparation. All authors have read and approved the final manuscript.

## References

[B1] Grunfeld C Feingold KR Metabolic disturbances and wasting in the acquired immunodeficiency syndrome N Engl J Med 1992 13 329 337 10.1056/NEJM199207303270506 1620172

[B2] Justman JE Hoover DR Shi Q Tan T Anastos K Tien PC Cole SR Hyman C Karim R Weber K Grinspoon S Longitudinal anthropometric patterns among HIV-infected and HIV-uninfected women J Acquir Immune Defic Syndr 2008 13 312 319 10.1097/QAI.0b013e318162f597 18197125 PMC4406344

[B3] Huang JS Harrity S Lee D Becerra K Santos R Mathews WC Body image in women with HIV: a cross-sectional evaluation AIDS Res Ther 2006 13 17 10.1186/1742-6405-3-17 16824226 PMC1553466

[B4] Wing RR Sinha MK Considine RV Lang W Caro JF Relationship between weight loss maintenance and changes in serum leptin levels Horm Metab Res 1996 13 698 703 10.1055/s-2007-979881 9013745

[B5] Ahima RS Lazar MA Adipokines and the peripheral and neural control of energy balance Mol Endocrinol 2008 13 1023 1031 10.1210/me.2007-0529 18202144 PMC2366188

[B6] De Rosa V Procaccini C Cali G Pirozzi G Fontana S Zappacosta S La Cava A Matarese G A key role of leptin in the control of regulatory T cell proliferation Immunity 2007 13 241 255 10.1016/j.immuni.2007.01.011 17307705

[B7] Steiner AA Romanovsky AA Leptin: at the crossroads of energy balance and systemic inflammation Prog Lipid Res 2007 13 89 107 10.1016/j.plipres.2006.11.001 17275915 PMC1976277

[B8] Sanchez-Pozo C Rodriguez-Bano J Dominguez-Castellano A Muniain MA Goberna R Sanchez-Margalet V Leptin stimulates the oxidative burst in control monocytes but attenuates the oxidative burst in monocytes from HIV-infected patients Clin Exp Immunol 2003 13 464 469 10.1111/j.1365-2249.2003.02321.x 14632752 PMC1808878

[B9] Grunfeld C Pang M Shigenaga JK Jensen P Lallone R Friedman J Feingold KR Serum leptin levels in the acquired immunodeficiency syndrome J Clin Endocrinol Metab 1996 13 4342 4346 10.1210/jc.81.12.4342 8954039

[B10] Yarasheski KE Zachwieja JJ Horgan MM Powderly WG Santiago JV Landt M Serum leptin concentrations in human immunodeficiency virus-infected men with low adiposity Metabolism 1997 13 303 305 10.1016/S0026-0495(97)90258-4 9054474 PMC3176667

[B11] Mynarcik DC Combs T McNurlan MA Scherer PE Komaroff E Gelato MC Adiponectin and leptin levels in HIV-infected subjects with insulin resistance and body fat redistribution J Acquir Immune Defic Syndr 2002 13 514 520 12473840 10.1097/00126334-200212150-00009

[B12] Kosmiski LA Bacchetti P Kotler DP Heymsfield SB Lewis CE Shlipak MG Scherzer R Grunfeld C Relationship of fat distribution with adipokines in human immunodeficiency virus infection J Clin Endocrinol Metab 2008 13 216 224 10.1210/jc.2007-1155 17940113 PMC2190751

[B13] Sanchez-Margalet V Martin-Romero C Gonzalez-Yanes C Goberna R Rodriguez-Bano J Muniain MA Leptin receptor (Ob-R) expression is induced in peripheral blood mononuclear cells by in vitro activation and in vivo in HIV-infected patients Clin Exp Immunol 2002 13 119 124 10.1046/j.1365-2249.2002.01900.x 12100031 PMC1906417

[B14] Macallan DC Noble C Baldwin C Jebb SA Prentice AM Coward WA Sawyer MB McManus TJ Griffin GE Energy expenditure and wasting in human immunodeficiency virus infection N Engl J Med 1995 13 83 88 10.1056/NEJM199507133330202 7777033

[B15] Macallan DC Noble C Baldwin C Foskett M McManus T Griffin GE Prospective analysis of patterns of weight change in stage IV human immunodeficiency virus infection Am J Clin Nutr 1993 13 417 424 8237855 10.1093/ajcn/58.3.417

[B16] Report on the global HIV/AIDS epidemic 2008: executive summary 2008 Geneva: Joint United Nations Programme on HIV/AIDS (UNAIDS)

[B17] Scott LE Glencross DK Monitoring reproducibility of single analysis, single platform CD4 cell counts in a high volume, low resource laboratory setting using sequential bead count rates Cytometry B Clin Cytom 2005 13 31 32 16100713 10.1002/cyto.b.20066

[B18] Collett D Modeling Survival Data in Medical Research Modeling Survival Data in Medical Research 2003 Chapman & Hall/CRC Press 83

[B19] Puoane T Steyn K Bradshaw D Laubscher R Fourie J Lambert V Mbananga N Obesity in South Africa: the South African demographic and health survey Obes Res 2002 13 1038 1048 10.1038/oby.2002.141 12376585

[B20] Azzoni L Chehimi J Zhou L Foulkes AS June R Maino VC Landay A Rinaldo C Jacobson LP Montaner LJ Early and delayed benefits of HIV-1 suppression: timeline of recovery of innate immunity effector cells AIDS 2007 13 293 305 10.1097/QAD.0b013e328012b85f 17255736

[B21] Koblavi-Deme S Maran M Kabran N Borget MY Kalou M Kestens L Maurice C Sassan-Morokro M Ekpini ER Roels TH Chorba T Nkengasong JN Changes in levels of immune activation and reconstitution markers among HIV-1-infected Africans receiving antiretroviral therapy Aids 2003 13 Suppl 3 S17 22 10.1097/00002030-200317003-00003 14565605

[B22] Madge S Kinloch-de-Loes S Mercey D Johnson MA Weller IV Lipodystrophy in patients naive to HIV protease inhibitors AIDS 1999 13 735 737 10.1097/00002030-199904160-00020 10397574

[B23] Grunfeld C Rimland D Gibert CL Powderly WG Sidney S Shlipak MG Bacchetti P Scherzer R Haffner S Heymsfield SB Association of upper trunk and visceral adipose tissue volume with insulin resistance in control and HIV-infected subjects in the FRAM study J Acquir Immune Defic Syndr 2007 13 283 290 10.1097/QAI.0b013e31814b94e2 18167644 PMC3164883

[B24] Shlay JC Visnegarwala F Bartsch G Wang J Peng G El-Sadr WM Gibert C Kotler D Grunfeld C Raghavan S Body composition and metabolic changes in antiretroviral-naive patients randomized to didanosine and stavudine vs. abacavir and lamivudine J Acquir Immune Defic Syndr 2005 13 147 155 10.1097/01.qai.0000143599.64234.15 15671799

[B25] Jain RG Furfine ES Pedneault L White AJ Lenhard JM Metabolic complications associated with antiretroviral therapy Antiviral Res 2001 13 151 177 10.1016/S0166-3542(01)00148-6 11448728

[B26] Carr A Samaras K Burton S Law M Freund J Chisholm DJ Cooper DA A syndrome of peripheral lipodystrophy, hyperlipidaemia and insulin resistance in patients receiving HIV protease inhibitors AIDS 1998 13 F51 58 10.1097/00002030-199807000-00003 9619798

[B27] Langford SE Ananworanich J Cooper DA Predictors of disease progression in HIV infection: a review AIDS Res Ther 2007 13 11 10.1186/1742-6405-4-11 17502001 PMC1887539

[B28] Appay V Sauce D Immune activation and inflammation in HIV-1 infection: causes and consequences J Pathol 2008 13 231 241 10.1002/path.2276 18161758

[B29] Sherman GG Scott LE Galpin JS Kuhn L Tiemessen CT Simmank K Meddows-Taylor S Meyers TM CD38 expression on CD8(+) T cells as a prognostic marker in vertically HIV-infected pediatric patients Pediatr Res 2002 13 740 745 12032270 10.1203/00006450-200206000-00013

[B30] Fitzgerald-Bocarsly P Jacobs ES Plasmacytoid dendritic cells in HIV infection: striking a delicate balance J Leukoc Biol 2010 13 609 620 10.1189/jlb.0909635 20145197 PMC2858309

[B31] Lucia B Jennings C Cauda R Ortona L Landay AL Evidence of a selective depletion of a CD16+ CD56+ CD8+ natural killer cell subset during HIV infection Cytometry 1995 13 10 15 10.1002/cyto.990220103 7587727

[B32] Tarazona R Casado JG Delarosa O Torre-Cisneros J Villanueva JL Sanchez B Galiani MD Gonzalez R Solana R Pena J Selective depletion of CD56(dim) NK cell subsets and maintenance of CD56(bright) NK cells in treatment-naive HIV-1-seropositive individuals J Clin Immunol 2002 13 176 183 10.1023/A:1015476114409 12078859

[B33] Arner P Regional differences in protein production by human adipose tissue Biochem Soc Trans 2001 13 72 75 10.1042/BST0290072 11356130

[B34] El-Sadr WM Mullin CM Carr A Gibert C Rappoport C Visnegarwala F Grunfeld C Raghavan SS Effects of HIV disease on lipid, glucose and insulin levels: results from a large antiretroviral-naive cohort HIV Med 2005 13 114 121 10.1111/j.1468-1293.2005.00273.x 15807717

[B35] Rose H Woolley I Hoy J Dart A Bryant B Mijch A Sviridov D HIV infection and high-density lipoprotein: the effect of the disease vs the effect of treatment Metabolism 2006 13 90 95 10.1016/j.metabol.2005.07.012 16324925

[B36] Scherzer R Shen W Bacchetti P Kotler D Lewis CE Shlipak MG Heymsfield SB Grunfeld C Simple anthropometric measures correlate with metabolic risk indicators as strongly as magnetic resonance imaging-measured adipose tissue depots in both HIV-infected and control subjects Am J Clin Nutr 2008 13 1809 1817 18541572 10.1093/ajcn/87.6.1809 PMC2587301

[B37] Fernandez-Riejos P Goberna R Sanchez-Margalet V Leptin promotes cell survival and activates Jurkat T lymphocytes by stimulation of mitogen-activated protein kinase Clin Exp Immunol 2008 13 505 518 10.1111/j.1365-2249.2007.03563.x 18234059 PMC2276975

[B38] Sanchez-Margalet V Martin-Romero C Santos-Alvarez J Goberna R Najib S Gonzalez-Yanes C Role of leptin as an immunomodulator of blood mononuclear cells: mechanisms of action Clin Exp Immunol 2003 13 11 19 10.1046/j.1365-2249.2003.02190.x 12823272 PMC1808745

[B39] Bullo M Garcia-Lorda P Peinado-Onsurbe J Hernandez M Del Castillo D Argiles JM Salas-Salvado J TNFalpha expression of subcutaneous adipose tissue in obese and morbid obese females: relationship to adipocyte LPL activity and leptin synthesis Int J Obes Relat Metab Disord 2002 13 652 658 10.1038/sj.ijo.0801977 12032749

[B40] Bullo M Garcia-Lorda P Megias I Salas-Salvado J Systemic inflammation, adipose tissue tumor necrosis factor, and leptin expression Obes Res 2003 13 525 531 10.1038/oby.2003.74 12690081

[B41] Herbein G Varin A Larbi A Fortin C Mahlknecht U Fulop T Aggarwal BB Nef and TNFalpha are coplayers that favor HIV-1 replication in monocytic cells and primary macrophages Curr HIV Res 2008 13 117 129 10.2174/157016208783884985 18336259

[B42] Schaffler A Scholmerich J Salzberger B Adipose tissue as an immunological organ: Toll-like receptors, C1q/TNFs and CTRPs Trends Immunol 2007 13 393 399 10.1016/j.it.2007.07.003 17681884

[B43] Popa C Netea MG Radstake TR van Riel PL Barrera P van der Meer JW Markers of inflammation are negatively correlated with serum leptin in rheumatoid arthritis Ann Rheum Dis 2005 13 1195 1198 10.1136/ard.2004.032243 15731289 PMC1755600

[B44] van Crevel R Karyadi E Netea MG Verhoef H Nelwan RH West CE van der Meer JW Decreased plasma leptin concentrations in tuberculosis patients are associated with wasting and inflammation J Clin Endocrinol Metab 2002 13 758 763 10.1210/jc.87.2.758 11836317

[B45] Arenzana-Seisdedos F Parmentier M Genetics of resistance to HIV infection: Role of co-receptors and co-receptor ligands Semin Immunol 2006 13 387 403 10.1016/j.smim.2006.07.007 16978874

